# Surgical Treatment for Occipital Condyle Fracture, C1 Dislocation, and Cerebellar Contusion with Hemorrhage after Blunt Head Trauma

**DOI:** 10.1155/2016/8634831

**Published:** 2016-10-05

**Authors:** Shigeo Ueda, Nobuhiro Sasaki, Miyuki Fukuda, Minoru Hoshimaru

**Affiliations:** Shin-Aikai Spine Center, Katano Hospital, Katano City, Osaka, Japan

## Abstract

Occipital condyle fractures (OCFs) have been treated as rare traumatic injuries, but the number of reported OCFs has gradually increased because of the popularization of computed tomography (CT) and magnetic resonance imaging (MRI). The patient in this report presented with OCFs and C1 dislocation, along with traumatic cerebellar hemorrhage, which led to craniovertebral junction instability. This case was also an extremely rare clinical condition in which the patient presented with traumatic lower cranial nerve palsy secondary to OCFs. When the patient was transferred to our hospital, the occipital bone remained defective extensively due to surgical treatment of cerebellar hemorrhage. For this reason, concurrent cranioplasty was performed with resin in order to fix the occipital bone plate strongly. The resin-made occipital bone was used to secure a titanium plate and screws enabled us to perform posterior fusion of the craniovertebral junction. Although the patient wore a halo vest for 3 months after surgery, lower cranial nerve symptoms, including not only neck pain but also paralysis of the throat and larynx, improved postoperatively. No complications were detected during outpatient follow-up, which continued for 5 years postoperatively.

## 1. Introduction

Head trauma can be accompanied by parenchymal brain damage; one study [1] reported that 4% of patients who experienced severe traumatic brain injuries, particularly with severely impaired consciousness (Glasgow Coma Scale scores of 3–6 on admission), developed occipital condylar fractures (OCFs). OCFs were considered rare traumatic injuries in the past, but, in recent years, they have been reported more frequently because of the popularization of computed tomography (CT) and magnetic resonance imaging (MRI) [1, 2]. OCFs rarely occurs sporadically, and, in many cases, they are associated with multiple traumas, especially cervical spine injury [3]. Patients exhibit various clinical symptoms of OCFs, but few are characteristic findings. However, it is known that OCFs lead to localized pain, limited range of motion, and severe nerve damage, especially lower cranial nerve (CN-IX to -XII) symptoms [4–6]. Clinical characteristics of lower cranial nerve palsy are hoarseness, difficulty swallowing (CN-IX and -X), and weakness in the shoulder and neck muscles (CN-XI). Hypoglossal nerve palsy (CN-XII) causes swallowing disturbance and masticatory dysfunction, as well as dysarthria and anarthria. Proper diagnosis and treatment are critical because coexisting symptoms can result from undiagnosed or untreated OCFs, which sometimes can be fatal [7].

We experienced a case in which a patient return to normal activities of daily living after surgical treatment who experienced dislocation of the atlas (C1), fractures of the left occipital condyle, and a traumatic hemorrhagic cerebellar contusion in the left hemisphere caused by blunt head trauma. An extensive literature review revealed that there have been few reports of surgical treatment in patients with OCFs. Therefore, this is the first report of the simultaneous performance of cranioplasty and posterior fusion of the craniovertebral junction.

## 2. Case Presentation

**Figure 1:**
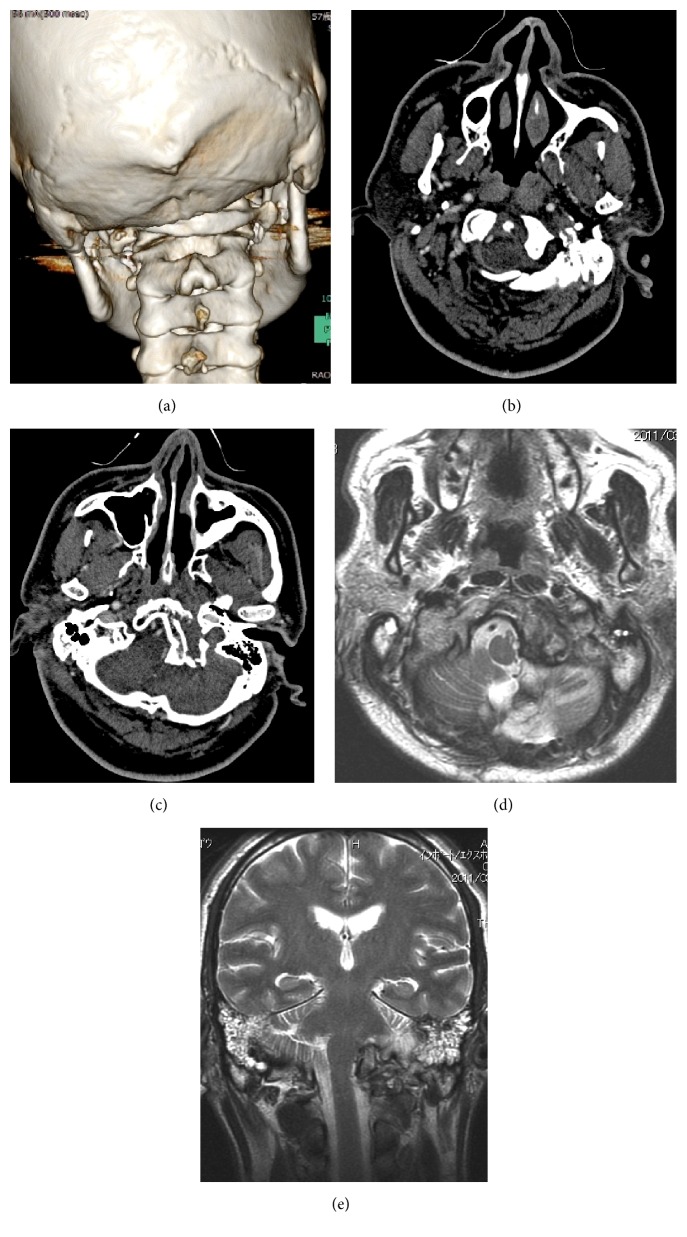
Images from a three-dimensional computed tomography (a), enhanced computed tomography (b and c), and magnetic resonance imaging (d and e) when the patient was first examined. The dislocated C1 that accompanied the occipital condyle fractures was intracranially impacted (a, b, and c). The left vertebral artery was not revealed by CT angiography (b and c). Cerebellar edema occurred due to traumatic hemorrhagic cerebellar contusions, which compresses the brainstem (d and e).

A 57-year-old man, who fell off of a bicycle and bruised the back of his head, became comatose immediately after the accident and was transported to a medical emergency center. He had a score of 7 on the GCS at the time of ambulance transport. CT of the head revealed hemorrhagic cerebellar contusion in the left hemisphere, fractures of the left occipital condyle, and C1 dislocation. Brain herniation occurred due to cerebral edema associated with the hemorrhagic contusion of the left cerebellar hemisphere, which was a medical emergency (Figure 1). Physicians at the medical emergency center performed lifesaving left suboccipital craniotomy and removed the hematoma and a part of the cerebellar hemisphere damaged by the contusion. Two weeks after surgery, the physicians intended to perform extubation due to the patient's improved of consciousness, since airway narrowing caused by left-sided vocal cord paralysis was observed, tracheostomy was additionally performed. At that time, neurological findings revealed lower cranial nerve (CN-IX to -XII) palsy. The patient was referred to our hospital for OCF and C1 dislocation treatment. On admission, he had clear consciousness but required bed rest in the supine position because of prominent neck pain that occurred when he was in the seated position. Although he complained of muscle weakness, which resulted from the prolonged bed rest, no obvious motor paralysis of the four extremities or sensory impairment was observed; however, he experienced left trapezius weakness, as well as paralysis of the larynx and the left side of the throat, which were associated with lower cranial nerve (CN-IX to -XII) palsy.

### 2.1. Imaging in the Preoperative Evaluation

**Figure 2:**
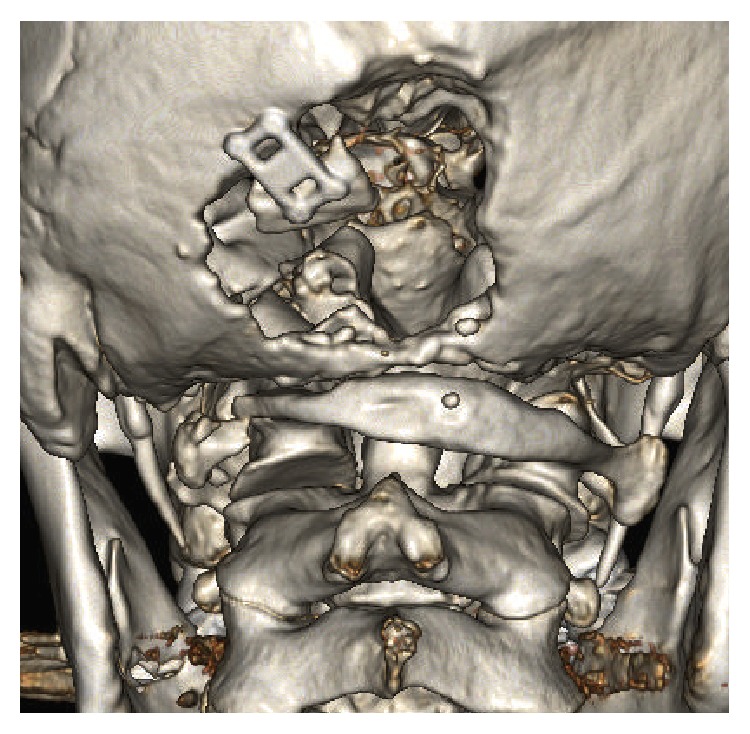
Computed tomography of the head and neck when the patient was referred to our hospital. Defects are observed in the left side and midline of the occipital bone.

CT revealed OCFs associated with C1 dislocation. A fracture extended to the left jugular foramen and hypoglossal canal. The left and medial sides of the occipital bone were defective because of the left suboccipital craniotomy (Figure 2). The left vertebral artery, which normally runs through the vertebrae proceeding away from the C1 vertebra, was not revealed by CT angiography, indicating traumatic vertebral artery occlusion.

### 2.2. Preoperative Care

We found that the neck pain was largely due to craniovertebral junction instability because halo vest immobilization (HVI) relieved the pain. We adjusted the craniovertebral angles in a halo vest and optimized them to prevent airway narrowing and swallowing disturbance. We made sure that HVI did not interfere with everyday activities for the patient after the halo vest had been in place and positioned at this angle for several days.

### 2.3. Surgery

**Figure 3:**
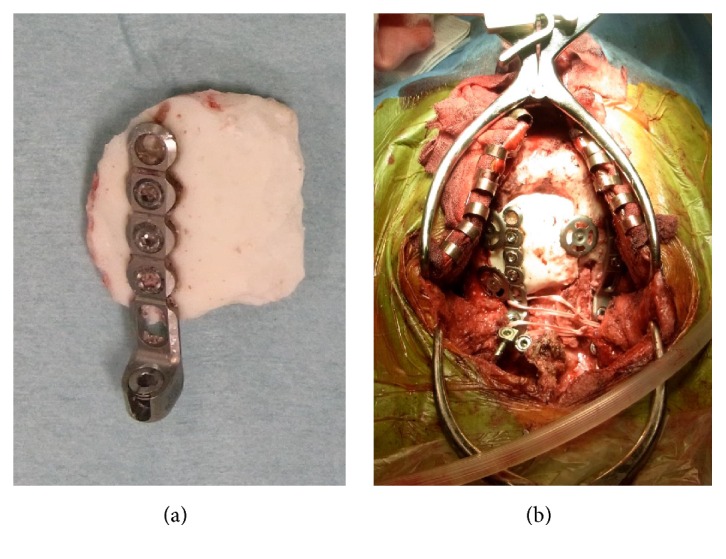
Intraoperative findings. A resin-made occipital bone was anchored with a titanium plate and titanium screws (a). Simultaneous cranioplasty and posterior fusion of the craniovertebral junction were performed. The iliac crest bone was grafted onto the occipital bone-C1-C2 alignment, and this grafted bone was fixed by a cable.

The patient was positioned prone in a halo ring after induction of general anesthesia. At the time of prone positioning, under lateral fluoroscopic guidance, we set the craniovertebral angles that had been optimized prior to surgery. We incised the skin along the marked median nuchal line from the external occipital protuberance to immediately above the seventh cervical (C7) spinous process and detached the posterior muscle group. A resin-made occipital bone was formed in accordance with the defective part of the occipital bone. Just before curing, the occipital bone plate and screws to be used for posterior fusion of the craniovertebral junction were also embedded together in resin. The resin-made occipital bone was placed in the defect's position and anchored with a titanium plate. We performed left-sided occipital cervical fusion by connecting vertebral arch pedicle screws with the occipital bone plate that had been secured to the resin-made occipital bone. On the right side, this occipital bone plate was immobilized with screws of C2 and C3 lateral mass at the remaining area of occipital bone. We harvested the iliac crest bone and grafted it onto the tip of the decorticalized spinous process of the C2 vertebra and the dorsal part of the occipital bone. The spongy bone was used in order to avoid creating dead space. The grafted bone was fixed by an ultrahigh molecular weight polyethylene cable (Figure 3).

### 2.4. Postoperative Care

**Figure 4:**
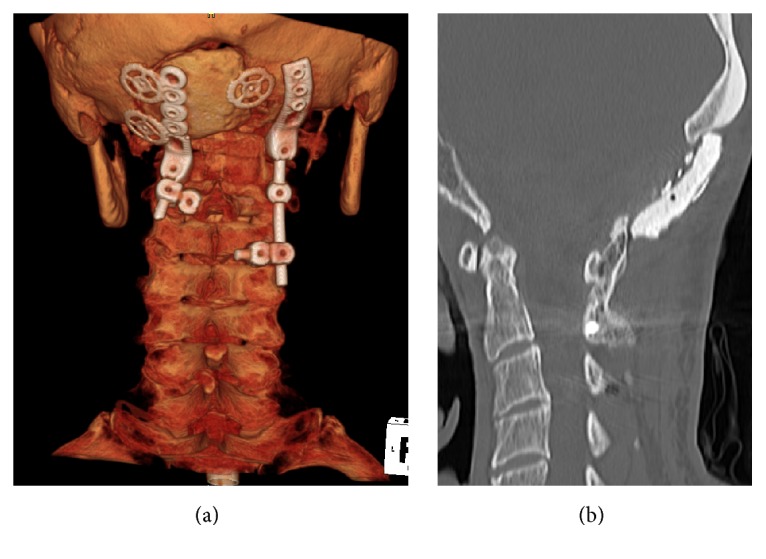
Clinical findings 50 months after surgery. Obvious implant breakage was not observed during a 5-year postoperative follow-up (a). The grafted bone was fused from the occipital bone through the C1 vertebra to the spinous process and the vertebral arch of the C2 vertebra (b).

The patient underwent rehabilitation in the halo vest for 3 months after surgery. Initially, he required tube feeding but later could ingest food orally because of physical therapy and dysarthria therapy offered by a speech therapist, as well as of swallowing and breathing training. He had mild paralysis of the left side of his throat and larynx, but sealing the tracheostomy site was possible. He walked independently and left the hospital following 5 months of rehabilitation after surgery. One year later, he returned to work as a school principal. Outpatient follow-up continued for 5 years postoperatively, but there were no newly developed complications. We observed trapezius weakness associated with left spinal accessory nerve palsy but did not detect symptoms of other lower cranial nerve palsies. X-ray and CT examination showed good bone graft incorporation, and no displacement of the resin-made occipital bone was observed (Figure 4).

## 3. Discussion

After head trauma, cervical spine injuries require careful evaluation, particularly craniovertebral junction injuries, such as OCFs, which can potentially lead to fatal outcomes or significant partial disability [8]. Furthermore, patients with severely impaired consciousness associated with intracranial injuries require careful attention, because stability of the craniovertebral junction is not always assessed and both management and treatment are not always adequately provided, which can lead to unfortunate outcomes [3, 9]. In our case, however, because the original imaging findings clearly showed C1 dislocation and strongly indicated craniovertebral junction instability, we strictly managed the patient, with local rest, in order to prevent cervical cord injury and following spinal shock when changing position. As a result, we successfully avoided secondary conditions.

In 1988, Anderson and Montesano published a classification system of OCFs [10], which was revised by Tuli et al. in 1997 [11]. In the revised classification system, Tuli et al. divided OCFs into three types based on the following approaches: with or without ligament injuries according to CT and MRI findings and with or without rotation and displacement of the occipital bone-C1-C2 alignment. The three types of this classification system are type 1, which includes fractures without displacement; type 2A, which includes fractures without ligament injuries; and type 2B, which includes a clearly identified ligament injury in the craniovertebral junction or identified rotation and displacement in occipital bone-C1-C2 alignment. Tuli et al. further explained that type 2B also includes potentially unstable fractures; therefore, we diagnosed our case as type 2B because it included C1 dislocation and indicated instability.

Because OCFs do not occur frequently, there has been no reported high-level evidence of a therapeutic strategy for OCFs [12]. Some reports have indicated that immobilization of the neck using a cervical collar and halo vest showed good outcomes when compared to those for patients without a medical device [3], but the sample sizes in these studies were small. Many healthcare institutions recommend using a cervical collar or a halo vest for about 6 to 12 weeks [9]. According to Tuli et al. [11], immobilization of the neck is unnecessary for type 1 fractures, but the use of a cervical collar is recommended for type 2A fractures, and the use of a halo vest or posterior fusion of the craniovertebral junction is recommended for type 2B fractures. Since our case was classified as type 2B, we needed to decide between HVI and posterior fusion of the craniovertebral junction. Surgical treatment was chosen because we estimated that the OCFs could be treated with external fixation using a halo vest, but the stability between two joints (O-C1 and C1-C2) was unlikely to be achieved due to the presence of C1 dislocation. In performing surgery, we considered decompression of the brainstem, jugular foramen, and hypoglossal canal, which is achieved by the removal of the dislocated fragments associated with C1 dislocation and OCFs. Likewise, there are also other reports indicating that the lower cranial nerve symptoms in patients experiencing OCFs complicated by lower cranial nerve palsy resolve after surgical removal of dislocated fragments [13, 14]; however there have only been three such cases, which is insufficient to show the superiority of surgical treatment [3]. On the other hand, removal of dislocated fragments from OCFs is associated with the risk of fatal complications, such as bleeding, caused by stroke and venous sinus injury induced by sigmoid sinus occlusion because of anatomical location close to the sigmoid sinuses, and so forth, and therefore requires careful consideration. There is also another report indicating that conservative management is effective in patients with brainstem compression caused by dislocated fragments [15].

In this case, removal of dislocated fragments, together with the reduction of C1 dislocation, was not performed because the patient had a history of traumatic cerebellar hemorrhage which indicated the risk of complications. Furthermore, although decompression was not performed, swallowing disturbance and dysarthria associated with lower cranial nerve palsy resolved during a follow-up period after posterior fusion. This clinical course also indicates that the removal of dislocated fragments may not always be essential for this type of injury. Additionally, a surgical approach for obtaining stability of the craniovertebral junction reportedly alleviates not only neurological fallout but also pain and actually provided effective pain relief in our patient.

A large number of surgical procedures for craniovertebral junction instability have been reported [16–18]. Many types of occipitocervical fusion systems are currently available, but most of them involve using an occipital bone plate placed in the thickest part of the midline of the occipital bone, which is an anatomically favorable way [19]. When this patient was transferred to our hospital, he had undergone suboccipital craniotomy, and a large skull defect was observed in the left side and midline of the occipital bone. We concluded that, in such a situation, it would be difficult to perform occipitocervical fusion, which requires sufficient occipital bone area and strength. Furthermore, it was difficult to establish the continuity of bone in left side of the occipital bone-C1-C2 because we dropped the plan to reduce C1 dislocation. We also thought that it would be difficult to provide enough stability of the craniovertebral junction by fixing only the right side of the occipital bone. We were further concerned about damaging the hardware after surgery because posterior fusion of the craniovertebral junction on only one side of the occipital bone would increase strain on the screws and rods. In order to solve all of the above issues, we performed cranioplasty by securing a resin-made occipital bone to the defective part using a titanium plate. We also performed occipital cervical fusion on the left side by connecting vertebral arch pedicle screws of the cervical spine with an occipital bone plate that had been secured to the resin-made occipital bone. These enabled us to establish strong stability of the craniovertebral junction on the left side of occipital bone. Therefore, to our knowledge, this is the first case report of the simultaneous performance of cranioplasty and posterior fusion of the craniovertebral junction. Postoperative CT showed good bone graft incorporation at the 5-year follow-up. Although our primary concern was the displacement of the resin-made occipital bone used for cranioplasty, it did not occur.
